# Physical Properties and Biochemical Composition of Extracellular Matrix-Derived Hydrogels Dictate Vascularization Potential in an Organ-Dependent Fashion

**DOI:** 10.1021/acsami.4c05864

**Published:** 2024-05-31

**Authors:** Meng Zhang, Fenghua Zhao, Yuxuan Zhu, Linda A. Brouwer, Hasse Van der Veen, Janette K. Burgess, Martin C. Harmsen

**Affiliations:** † Department of Pathology and Medical Biology, University Medical Center Groningen, University of Groningen, Hanzeplein 1 (EA11), Groningen 9713 GZ, The Netherlands; ‡ University Medical Center Groningen, W.J. Kolff Institute for Biomedical Engineering and Materials Science-FB41, University of Groningen, A. Deusinglaan 1, Groningen 9713 AV, The Netherlands; § University Medical Center Groningen, Department of Biomedical Engineering-FB40, University of Groningen, A. Deusinglaan 1, Groningen 9713 AV, The Netherlands; ∥ Department of Computer Science, Rensselaer Polytechnic Institute, Troy, New York 12180, United States; ⊥ University Medical Center Groningen, Groningen Research Institute for Asthma and COPD (GRIAC), University of Groningen, Hanzeplein 1 (EA11), Groningen 9713 AV, The Netherlands

**Keywords:** vascularization, extracellular matrix, ECM hydrogel, endothelial cells, biomechanics

## Abstract

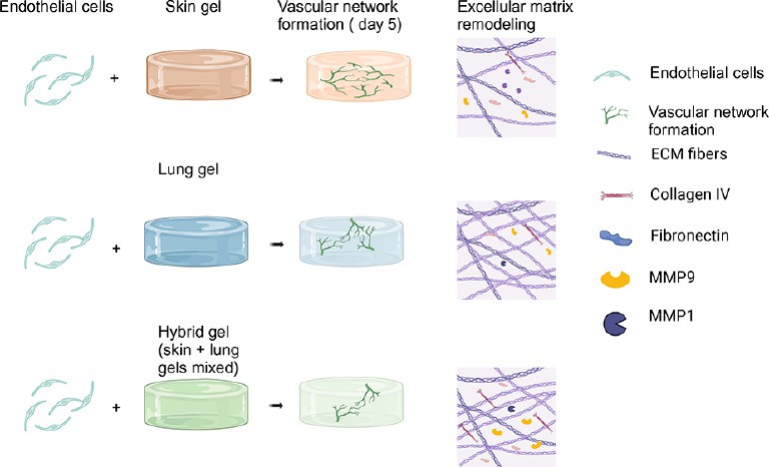

The inherent extracellular matrix (ECM) originating from a specific tissue impacts the process of vascularization, specifically vascular network formation (VNF) orchestrated by endothelial cells (ECs). The specific contribution toward these processes of ECM from highly disparate organs such as the skin and lungs remains a relatively unexplored area. In this study, we compared VNF and ECM remodeling mediated by microvascular ECs within gel, lung, and combinations thereof (hybrid) ECM hydrogels. Irrespective of the EC source, the skin-derived ECM hydrogel exhibited a higher propensity to drive and support VNF compared to both lung and hybrid ECM hydrogels. There were distinct disparities in the physical properties of the three types of hydrogels, including viscoelastic properties and complex architectural configurations, including fiber diameter, pore area, and numbers among the fibers. The hybrid ECM hydrogel properties were unique and not the sum of the component ECM parts. Furthermore, cellular ECM remodeling responses varied with skin ECM hydrogels promoting matrix metalloproteinase 1 (MMP1) secretion, while hybrid ECM hydrogels exhibited increased MMP9, fibronectin, and collagen IV deposition. Principal component analysis (PCA) indicated that the influence of a gel’s mechanical properties on VNF was stronger than the biochemical composition. These data indicate that the organ-specific properties of an ECM dictate its capacity to support VNF, while intriguingly showing that ECs respond to more than just the biochemical constituents of an ECM. The study suggests potential applications in regenerative medicine by strategically selecting ECM origin or combinations to manipulate vascularization, offering promising prospects for enhancing wound healing through pro-regenerative interventions.

## Introduction

Regeneration of organs and tissues after damage demands vascularization to reinstate adequate perfusion, which is essential to facilitate the exchange of gas, nutrients, and waste, as well as to enable the recruitment and influx of immune cells.^1−3^ Upon acute skin damage, wound healing depends on the proliferation and migration of resident mesenchymal cells and proper vascularization.^4^

Traditional therapeutic approaches face several obstacles when it comes to the vascular regeneration treatment of skin injury. One of the challenges is the biocompatibility of applied materials that should support healing.^5^ Also, traditional treatment may not adequately stimulate or support endogenous vascular formation.^6^ To address these limitations, novel approaches, such as biomaterials or regenerative medicine, are being explored to promote targeted and effective vascularization.

The ECM hydrogel derived from natural tissues is a promising biomaterial for directing vascularization by endothelial cells. Natural ECM hydrogels facilitate tissue reconstruction in vivo,^7−9^ while porcine ECM hydrogels are biocompatible and augment skin wound healing in rats through upregulated vascularization.^10^ It appears that ECM hydrogels preserve the biochemical complexity, nanostructure, and biological inductive properties inherent in the native matrix.^11^ ECM hydrogels derived from different organs show a large overlap in biochemical composition.^12,13^ However, despite the similarities in the bulk biochemical composition among organ-derived ECM hydrogels, each hydrogel still possesses distinct characteristics associated with its organ source. For instance, the lung-decellularized ECM hydrogel lacks glycosaminoglycans (GAGs), while conversely, these were found to be relatively higher in the skin ECM hydrogel. Moreover, the collagen I content in the lung ECM hydrogel was observed to be lower compared with the skin ECM hydrogel. These variations extend beyond biochemical composition and encompass differences in mechanical properties, including stiffness and viscosity, as well as in the fibrous microstructure.^12^ The specific biological consequences of these variances remain to be elucidated. The manner in which these diverse ECM hydrogels impact fundamental processes occurring in tissues and organs, such as vascularization, remains a topic to be explored.

In the preceding decades, significant attention has been dedicated to the vascularization process from the standpoint of endothelial cells. However, there remains limited understanding regarding the influence of the origin and physical characteristics of ECM.^14^ Therefore, we set out to explore differences in vascularization and ECM remodeling between skin ECM hydrogel and lung ECM hydrogel and tested the hypothesis that equally mixed skin and lung ECM hydrogels (referred to as “hybrid ECM hydrogel” hereafter) improve vascularization in vitro.

## Materials and Methods

### Hydrogel Synthesis

The porcine skin and lung were purchased from a slaughterhouse (Kroon Vlees, Groningen, The Netherlands). The generation of decellularized ECM was performed as described previously.^12,15^ In short, skin tissue was dissected (1 cm^3^) and combined with ice-cold Dulbecco’s phosphate-buffered saline (DPBS) (Lonza Walkersville, Inc., Walkersville, MD, USA) before being finely divided in a kitchen blender (Bourgini, Breda, The Netherlands) with DPBS to create a paste. This tissue paste was subjected to sonication using an ultrasonic homogenizer (Sigma-Aldrich, Amsterdam, The Netherlands) at 100% power for 1 min. The paste was then washed with DPBS twice and separated by centrifugation at 3000 *g* until the supernatant was transparent. Subsequent incubation occurred with 0.05% trypsin in DPBS (Thermo Fisher Scientific, Waltham, MA, USA) at 37 °C with consistent shaking for 4 h. After two further washes with PBS, the slurry underwent overnight incubation in Milli-Q water at 37 °C with continuous agitation. Next, the tissue homogenate was treated with excess saturated NaCl (6M) for 3 h, 1% SDS, 1% Triton X-100, and 1% sodium deoxycholate in Milli-Q water, along with 30 μg/mL DNase (Roche Diagnostics GmbH, Mannheim, Germany) in 1.3 mM MgSO_4_ and 2 mM CaCl_2_. These incubations were maintained under shaking at 37 °C overnight. Between treatments, the homogenate was washed three times with Milli-Q water. Finally, the homogenate was washed with DPBS six times and then replaced with 70% ethanol for overnight sterilization at room temperature.

The lung tissue was dissected (1 cm^3^), with cartilaginous airways and large blood vessels removed. The remaining procedure was identical except that the lung homogenate underwent two rounds of treatment: 0.1% Triton X-100, 2% sodium deoxycholate, 1 M NaCl solution at 37 °C with shaking, followed by 30 μg/mL DNase in 1.3 mM MgSO_4_ and 2 mM CaCl_2_, 10 mM Tris pH 8 at 4 °C with constant shaking. The skin and lung ECM samples were frozen in liquid nitrogen and lyophilized with a freeze-dryer (Labconco, Kansas City, MO, USA), and then ground into a fine powder using an ULTRA-TURRAX (IKA, Staufen, Germany).

To prepare hydrogels, 20 mg/mL of ECM powder was digested with 2 mg/mL of porcine pepsin (Sigma-Aldrich, St. Louis, MO, USA) in 0.01 M HCl under constant agitation at room temperature; the skin ECM powder required 24 h of digestion and the lung ECM powder 48 h. Postdigestion, the ECM was neutralized by adding 1/10th volume of 0.1 M NaOH and subsequently 1/10th volume of 10xDPBS, forming an isotonic, neutral pH ECM pregel, stored at 4 °C until use.

### 3D Cell Culture

Human pulmonary microvascular endothelial cells (HPMEC-ST1.6R^16^ HPMEC in the text), Johannes Gutenberg University, Mainz, Germany) and human microvascular endothelial cells (HMEC-1,^17^ HMEC in the text) were retrovirally tagged with EGFP (green fluorescence) by third-generation VSV-pseudotyped replication-deficient lentiviruses.^18^ HPMEC were cultured in an endothelial-specific growth medium composed of RPMI-1640 (obtained from Lonza, Basel, Switzerland), supplemented with 20% heat-inactivated fetal bovine serum (FBS, sourced from Sigma-Aldrich, MO, United States), 1% penicillin/streptomycin (product no. 15140122, procured from Gibco Invitrogen, Carlsbad, CA, USA), 1% l-glutamine (catalog #17-605E, provided by Lonza BioWhittaker, Verviers, Belgium), 5 U/mL heparin (manufactured by LEO Laboratories Limited, Ballerup, Denmark), and 20 μg/mL endothelial growth factors (EGF, derived from bovine brain extract^19^). HMEC were cultured in MCDB 131 medium (supplied by Gibco, Carlsbad, CA, USA) containing 10% FBS, 10 mM l-glutamine, 10 ng/mL EGF, and 1 μg/mL hydrocortisone (Sigma, MO, United States). 0.5 × 10^6^ HPMEC or HMEC were suspended in 10 μL of the culture medium which was carefully and homogeneously mixed with 200 μL of skin or lung or skin and lung ECM pregel mixed in 1:1 (100 μL for each type of pregel). The cell-gel mixtures were cast into single wells of 48-well plates and incubated at 37 °C for 45 min to solidify the gel. Subsequently, 500 μL of endothelial culture medium was added to the wells. Hydrogels without cells were used as the controls.

### Fluorescence Cell Imaging

After 5 days of culturing at 37 °C 5% CO_2_, inverted fluorescence microscopy (EVOS model M5000, Thermo Fisher) was used to acquire fluoromicrographs to visualize the vascular-like network formation (VNF) by HPMEC and HMEC. Both HPMEC and HMEC were visualized using GPF “light cubes” (λ^ex^/λ^em^ 470/510 nm). VNF was further processed with the endothelial tube formation assay—angiogenesis analyzer in Fiji.^20^ The micrographs compressed the original 3D VNF onto a 2D plane, which displaced genuine branched tubes and tubes that crossed each other at different planes in the gel. Because no suitable 3D imaging and quantification software was available to analyze our images, we decided to process all images in this way.

### Characterization of the Mechanical Properties

The gels loaded with cells were subjected to uniaxial compression at three locations using a 2.5 mm plunger using a low-load compression tester (LLCT) and 20% compression (0.2 strain) in 1 s.^12,21^ The compression sites were positioned at least 2 mm away from the edge of the gel and were separated by 2 mm or more from each other. The stress relaxation test was conducted under “wet” mode and at room temperature. The LLCT load cell and linear positioning for control and data were acquired using LabVIEW 7.1 software.^22^ During compression, the increase in stress was continuously monitored to derive the elastic modulus from the stress–strain curve’s slope. Upon reaching a strain of 0.2, it was maintained at this level for 100 s, while continuously monitoring the stress. The percentage of stress relaxation was calculated by comparing the stress at *t* = 0 and 100 s.

### Hydrogel Ultrastructure

The hydrogel ultrastructure was examined by using scanning electron microscopy (SEM). After culturing, the fixation of hydrogels and the sample preparation for SEM were performed, as described in previous published research.^15^ In short, hydrogels underwent fixation using a solution composed of 2.5% glutaraldehyde (111-30-8, Sigma, Darmstadt, Germany) and 2% paraformaldehyde in phosphate-buffered saline (PBS) at 4 °C for a duration of 24 h. The hydrogels underwent three washes with Dulbecco’s phosphate-buffered saline (DPBS) and one wash with Milli-Q water to eliminate any residual fixatives and salts. Following this, the samples were subjected to dehydration and embedding in paraffin. The resultant 50 μm thick sections were sliced and affixed onto glass coverslips measuring 18 × 18 mm. The sections underwent deparaffinization in xylene, followed by rehydration through a graded series of ethanol concentrations (100, 96, and 70%). The desiccated slides were affixed to 6 mm scanning electron microscopy (SEM) pin stubs (Agar Scientific, Stansted, UK) and coated with carbon using a Leica EM ACE600 sputter coater device (Leica Microsystems B.V., Amsterdam, The Netherlands). The hydrogels were visualized at magnifications of 5000, 10,000, and 25,000× (respectively 5, 10, and 25K) operating at 3 kV using the Zeiss Supra 55 scanning electron microscope (Carl Zeiss NTS GmbH). Fibers surrounding the cells were assessed “25K” micrographs using the Diameter J plugin in Fiji.^23^

### Immunofluorescence Staining

Thin paraffin sections (4 μm) were deparaffinized and rehydrated. For antigen retrieval, slides were incubated in 10 mM citric acid (pH 6) at 85 °C overnight. Slides were washed with demi water and PBS and subsequently blocked in 4% BSA for 15 min at room temperature. Afterward, the slides were incubated for 1 h with the first antibody (Table 1) at room temperature. After that, the slides were washed with PBS three times and incubated with a secondary antibody (Table 2) for 1 h. Opal 650 (Akoya Biosciences, 1:200) was used for fibronectin to amplify the signal. Opal 650 diluted in 0.1 M borate buffer with 0.003% hydrogen peroxide (Merck, Darmstadt, Germany) and incubated with slides for 15 min. The slides were washed with demi water 3 times and incubated with DAPI (Merck 1:5000) for 10 min. These staining images were generated by a SP8 confocal microscope (Leica, Wetzlar, Germany).

**Table 1:** Information on the First Antibody

primary antibody	host	company	concentration
fibronectin	rabbit	ab6584, Abcam	1:100
Ki67	rabbit	ab211536, Abcam	1:300
MMP1	rabbit	ab52631, Abcam	1:100
MMP9	rabbit	MA5-15886, Thermo Fisher	1:100
collagen IV	goat	1340-01, Southern Biotech	1:100

**Table 2:** Information of the Second Antibody

second antibody	corresponding first antibody	host	company	concentration
immunoglobulins/HRP	fibronectin	goat	P0448, Dako	1:200
Alexa Fluor 647	Ki67	donkey	A31573, Invitrogen	1:300
Alexa Fluor 647	MMP1	donkey	A31573, Invitrogen	1:300
Alexa Fluor 647	MMP9	donkey	A31573, Invitrogen	1:300
Alexa Fluor 555	collagen IV	rabbit	A21431, Invitrogen	1:300

The staining images obtained were analyzed with CellProfiler v 4.2.5. For each sample, a total of three images (*n* = 3) were randomly selected to quantify the expression levels of MMP1, MMP9, fibronectin, and collagen IV, as well as the number of nuclei (i.e., cells) per image. The quantification of protein expression per cell was determined by dividing the overall protein expression by the number of nuclei observed in each image. Additionally, the ratio of Ki67 positive nuclei was calculated as the fraction of the number of positive Ki67 nuclei divided by the total number of nuclei to obtain the proliferation index.

### Principal Component Analysis

PCA was performed on a data set that records material property and VNF, to explore the correlation between the extent of tube formation and material characteristics. PCA diminishes the data set’s dimensionality by identifying a reduced set of variables that retain most of the information present in the larger set.

In our experiment, the data matrix X ∈ R50 × 20, which consisted of 50 samples and 20 features. All variables were normalized using Min–Max normalization, scaling the data to a specified range (default: 0–1) to mitigate the influence of scale differences between features on the PCA algorithm. The PCA algorithm was implemented using the sklearn library in Python.^24^ By observation of the scatter plots of the samples after performing PCA, patterns and trends in the samples could be identified. If there is a clear linear relationship between the original features and the PCA results, then a high correlation will exist between the original features and the PCA results. To achieve consistency in the positive and negative values of PC1, PC2, and PC3, where positive values indicate a positive impact on the VNF, we standardize by assigning the opposite numerical values to all components of PC1 and PC3. A higher PC value signifies a more substantial positive contribution to the VNF.

### Statistical Analysis

All statistical analyses were conducted using GraphPad Prism v9.2.0 (GraphPad Company, San Diego, CA, USA). Prior to analysis, all data underwent outlier detection utilizing the robust regression and outlier removal (ROUT) test. Subsequently, one-way analysis of variance (ANOVA) was employed for data analysis. Significance was determined at the *p* < 0.05 level in the respective statistical tests. Components with absolute PC values in PCA exceeding the median are deemed significant for VNF.

## Results

### Skin-Derived ECM Hydrogel Enhances VNF by ECs Irrespective of Their Organ-Specific Origin

**Figure 1:**
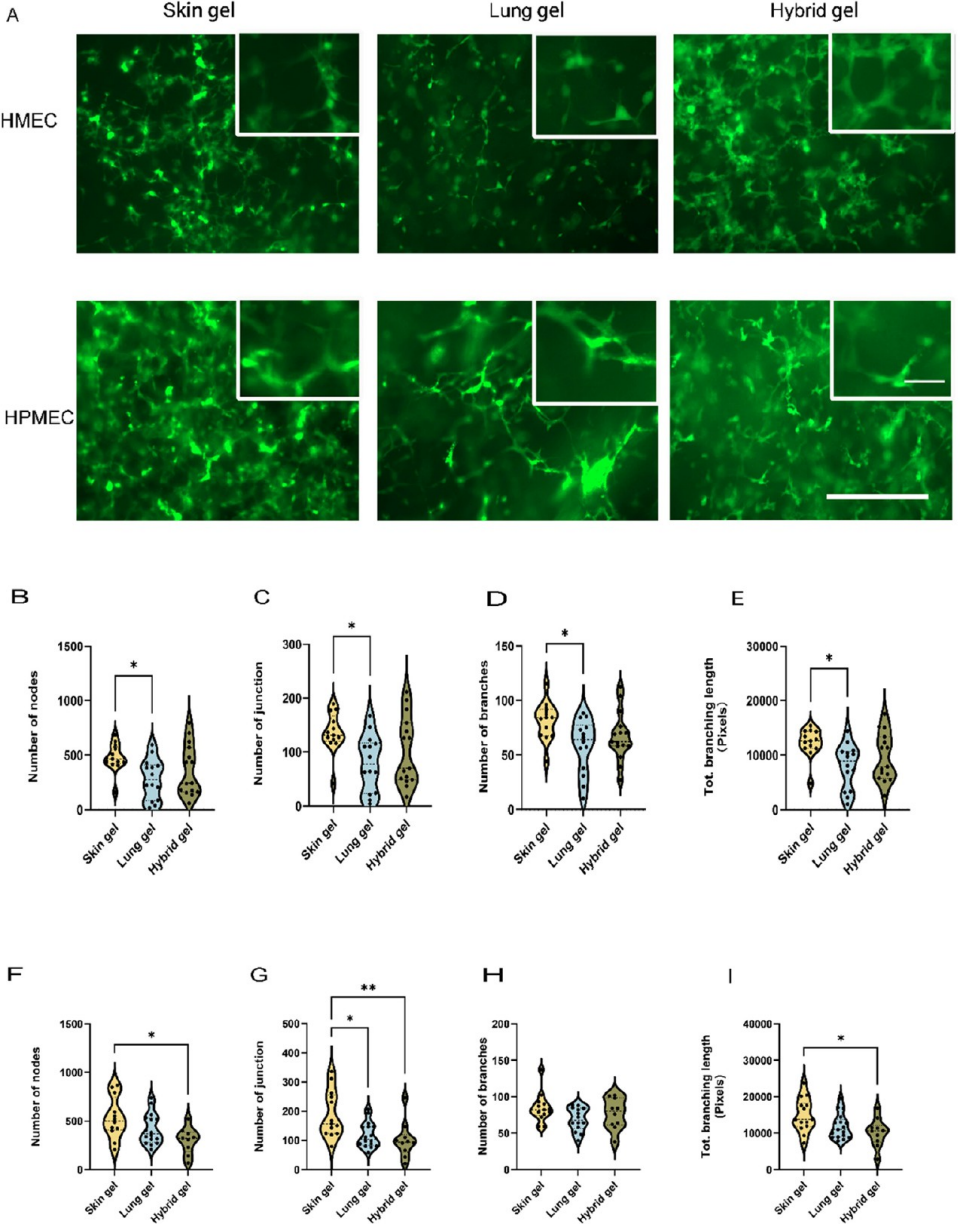
VNF by ECs was visualized in three different ECM hydrogels. (A) EGFP-expressing HMECs or HPMECs (green) were seeded in skin, lung, and hybrid hydrogels in 48-well plates and cultured for 5 days. Scale bar: 400 μm. (B) Quantification of nodes formed by HMEC in three distinct hydrogel types was done with FIJI software. (C) Quantification of the number of master junctions formed by HMEC in three distinct hydrogel types. (D) Quantification of the number of branches produced by HMEC in three distinct hydrogel types. (E) Quantification of total branching length generated by HMEC in three distinct hydrogel types. (F) Quantification of the number of nodes produced by HPMEC in three distinct hydrogel types. (G) Quantification of the number of master junctions produced by HPMEC in three distinct hydrogel types. (H) Quantification of the number of branches produced by HPMEC in three distinct hydrogel types. (I) Quantification of total branching length produced by HPMEC in three distinct hydrogel types. The data are from 7 independent experiments. Three different random regions of interest (ROI) were measured for every single sample, and each dot represents a measurement of a randomized region. One-way ANOVA comparing gels, *p < 0.05, **p < 0.01.

Spontaneous VNF and formation of branched and extensive vascular-like structures by HMEC and HPMEC were observed in all three distinct ECM hydrogels after 5 days of culture (Figure 1A, green).

The VNF by HMEC in skin ECM hydrogels was consistently higher than that in lung ECM hydrogels for all four measured parameters (Figure 1B–E). The analysis of VNF formed by ECs was delineated as illustrated in Suppl. Figure S1A. Specifically, the number of nodes was 477.2 ± 129.4 in skin ECM hydrogels and 279.9 ± 184.3 in lung ECM hydrogels (*p* = 0.0269); the number of junctions was 135.6 ± 37.35 in skin ECM hydrogels and 79.4 ± 51.8 in lung ECM hydrogels (*p* = 0.0224). Moreover, the number of branches and the total branching length were 80.9 ± 17.5 and 12,255 ± 2681 pixels in skin ECM hydrogels, respectively, whereas in lung ECM hydrogels, these values were lower, respectively, 58.2 ± 24.5 (*p* = 0.0267) and 7897 ± 4089 pixels (*p* = 0.0150). The VNF by HMEC in hybrid ECM hydrogels did not differ from skin ECM hydrogels or lung ECM hydrogels due to the large variation in responses (Figure 1B–E).

Like VNF by HMEC, VNF by HPMEC (Figure 1F–I) was also the highest in skin ECM hydrogels, but notably, this was only in comparison to hybrid ECM hydrogels. while only the number of junctions, but not the number of branches or total branching length, in lung ECM hydrogels was lower than in skin ECM hydrogels. The number of HPMEC-generated nodes was 532.3 ± 213.1 in skin ECM hydrogels and 299.8 ± 136.6 in hybrid ECM hydrogels (*p* = 0.0123), and the reduced number of junctions was 188.6 ± 80.6 in skin ECM hydrogels and 101.3 ± 61.8 in hybrid ECM hydrogels (*p* = 0.0057).

### Proliferation of HPMEC Is Highest in Skin ECM Hydrogels after 5 Days of Culturing

**Figure 2:**
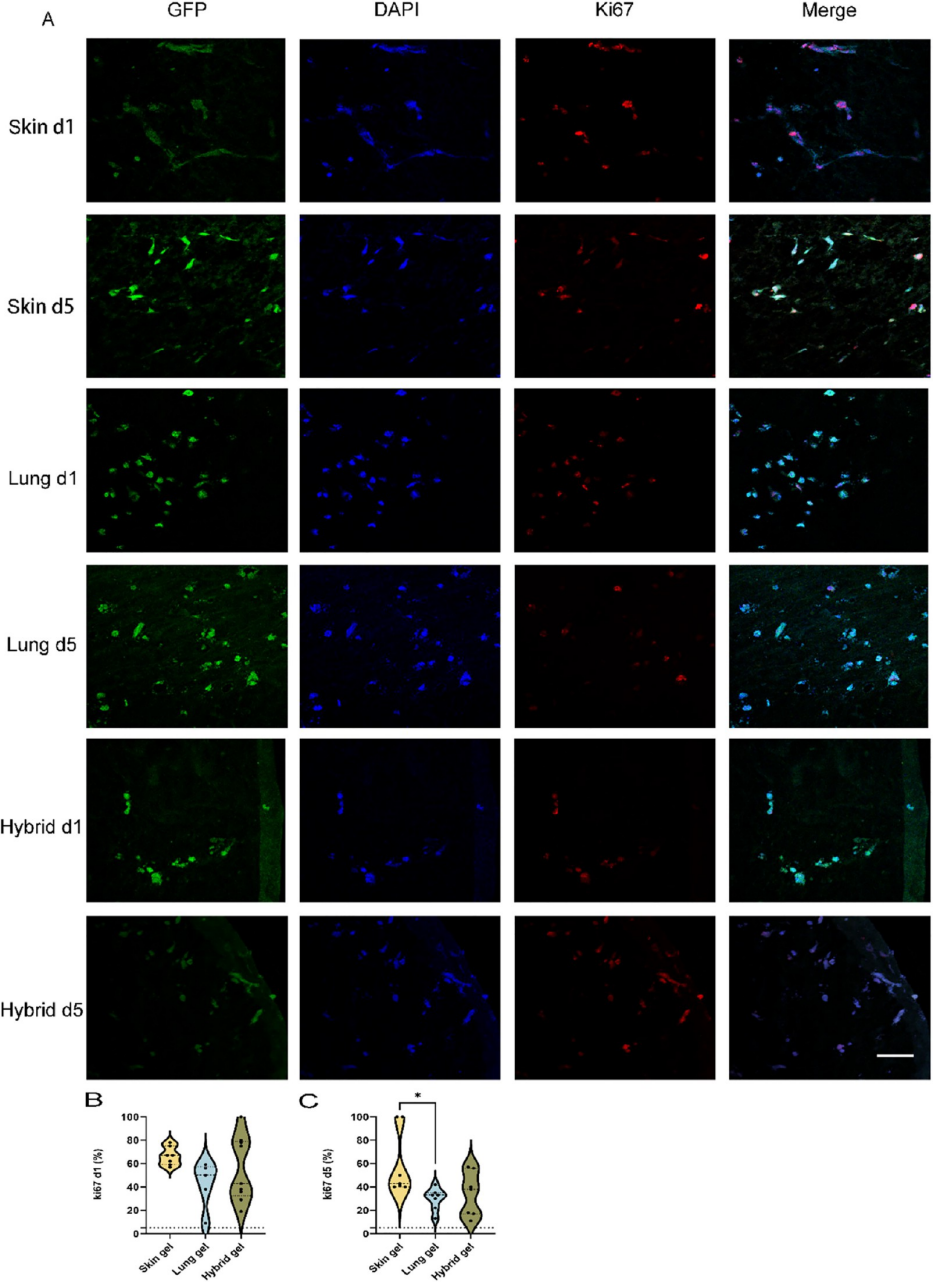
Comparison of Ki67 expression in HPMEC seeded in three distinct ECM hydrogels. (A) Representative fluoromicrographs of 4 μm sections of paraffin-embedded skin, lung, and hybrid ECM hydrogels loaded with HPMEC. Merged images: green, GPF-labeled HPMEC; red, Ki67; blue, nuclei (DAPI). Scale bars: 58 μm. (B) Comparison of the percentage of Ki67 positive nuclei in the three different gels at day 1. (C) Comparison of the percentage of Ki67 positive nuclei in the three different gels on day 5.

Considering that (irrespective of gel type) HMEC and HPMEC had similar VNF patterns but the HPMEC was slightly higher, we continued our experiments with HPMEC only. Fluorescence staining was performed on sections of paraffin-embedded skin, lung, and hybrid ECM hydrogels containing HPMEC. HPMEC had elongated in the skin ECM hydrogels within 1 day (Figure 2A, top row, green), whereas at day 1, cells remained rounded in the lung and hybrid ECM hydrogels. At day 1 and irrespective of gel type, approximately 50% of HPMEC were proliferating as judged by the Ki67 expression (Figure 2B). Ki67 serves as a biomarker of proliferation, utilized to quantify the growth fraction within a cell population.^25^ Albeit, skin ECM hydrogels tended to promote proliferation more than lung or hybrid ECM hydrogels, or alternatively, lung and hybrid ECM hydrogels tended to inhibit proliferation more. These differences were maintained at 5 days when the percentage proliferation of HPMEC in skin ECM hydrogels was higher than in lung ECM hydrogels (Figure 2C, 59% ± 28% vs 30% ± 9%, *p* = 0.0378). The variation in the proliferation of HPMEC in hybrid ECM hydrogels was too large and did not differ from that of either skin or lung ECM hydrogels.

The data are from three independent experiments. Three random ROIs were measured for every single sample, and each dot represents a measurement of a randomized region. One-way ANOVA comparing gel, *p < 0.05.

### Physical Properties of Different Organ-Derived ECM Hydrogels Change during VNF

**Figure 3:**
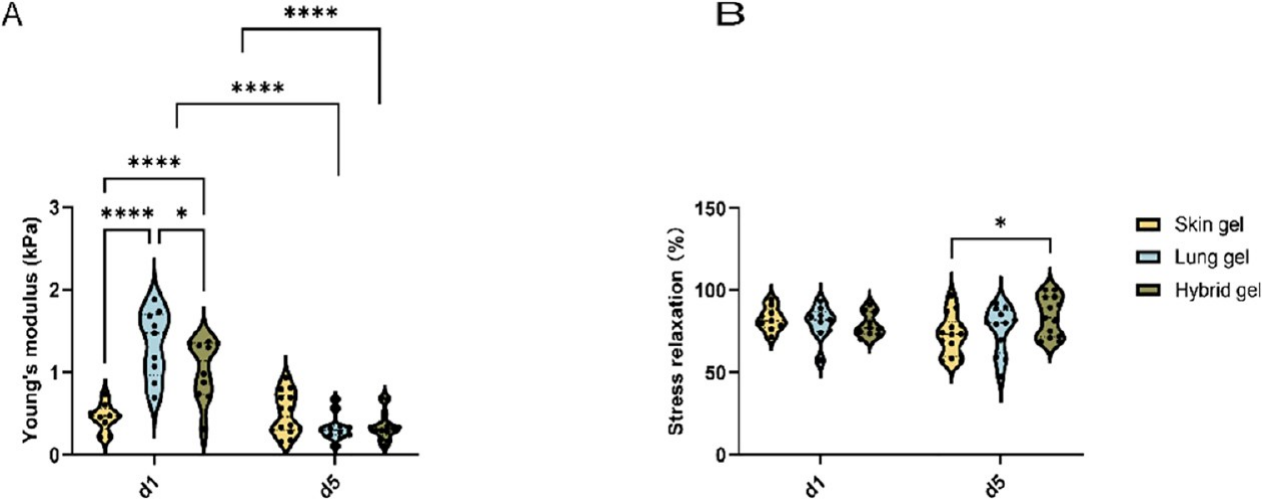
Comparison of the physical characteristics of three ECM hydrogels. (A) Stiffness of skin, lung, and hybrid ECM hydrogels loaded with HPMEC at days 1 and 5. (B) Total stress relaxation of skin, lung, and hybrid ECM hydrogel was loaded with HPMEC at days 1 and 5. After compressing the skin ECM hydrogel for 20%, the stress relaxation was recorded for 100 s.

Hydrogels’ stiffness and stress relaxation were measured using a low-load compression tester (LLCT). Both were determined after 20% strain and relaxation for 100 s. The stiffness of the cell-free skin ECM hydrogels was lower than lung ECM hydrogels (Suppl. Figure S1A). At 24 h postseeding, the stiffness of the skin ECM hydrogels was lower than lung ECM hydrogels (Figure 3A, 0.45 ± 0.16 vs 1.35 ± 0.41 kPa, *p* < 0.0001) and hybrid ECM hydrogels (0.45 ± 0.16 vs 1.03 ± 0.37 kPa, *p* = 0.0025). After 5 days of culture, the stiffness of the HPMEC-loaded gels did not differ, irrespective of the hydrogel origin. It was observed that both the lung and hybrid ECM hydrogels exhibited a reduction in firmness compared to their initial state on day 1, a change not observed in the skin ECM hydrogel (Figure 3A, *p* < 0.0001).

Hydrogels also comprise a viscous component that dictates stress relaxation. Cell-free skin ECM hydrogel reached close to 100% stress relaxation, which was higher than cell-free lung and hybrid ECM hydrogel (Suppl. Figure S1B). However, this difference disappeared in HPMEC-seeded hydrogels already at day 1 (Figure 3B). Prolonged culturing (5 days) decreased the stress relaxation of the skin ECM hydrogel compared to the hybrid ECM hydrogel (72.8% ± 12.5% vs 84.0% ± 12.1%, *p* = 0.0322).

Differential viscoelastic properties are discernible among various organ-derived hydrogel substrates. The skin ECM hydrogel was softer than the other two hydrogels, which may contribute to the augmentation of VNF. Conversely, the activities of ECs, including vascularization and proliferation, lead to significant modifications in the physical attributes and physical characteristics of ECM.

The data are from three independent experiments. Three randomly selected ROIs were measured for every single sample, and each dot represents a measurement of a randomized region. Tukey’s multiple comparisons test, *p < 0.05, **p < 0.01, ****p ≪ 0.0001.

### Ultrastructure of Hydrogels Depends on the Organ Origin and Cellular Influence

**Figure 4:**
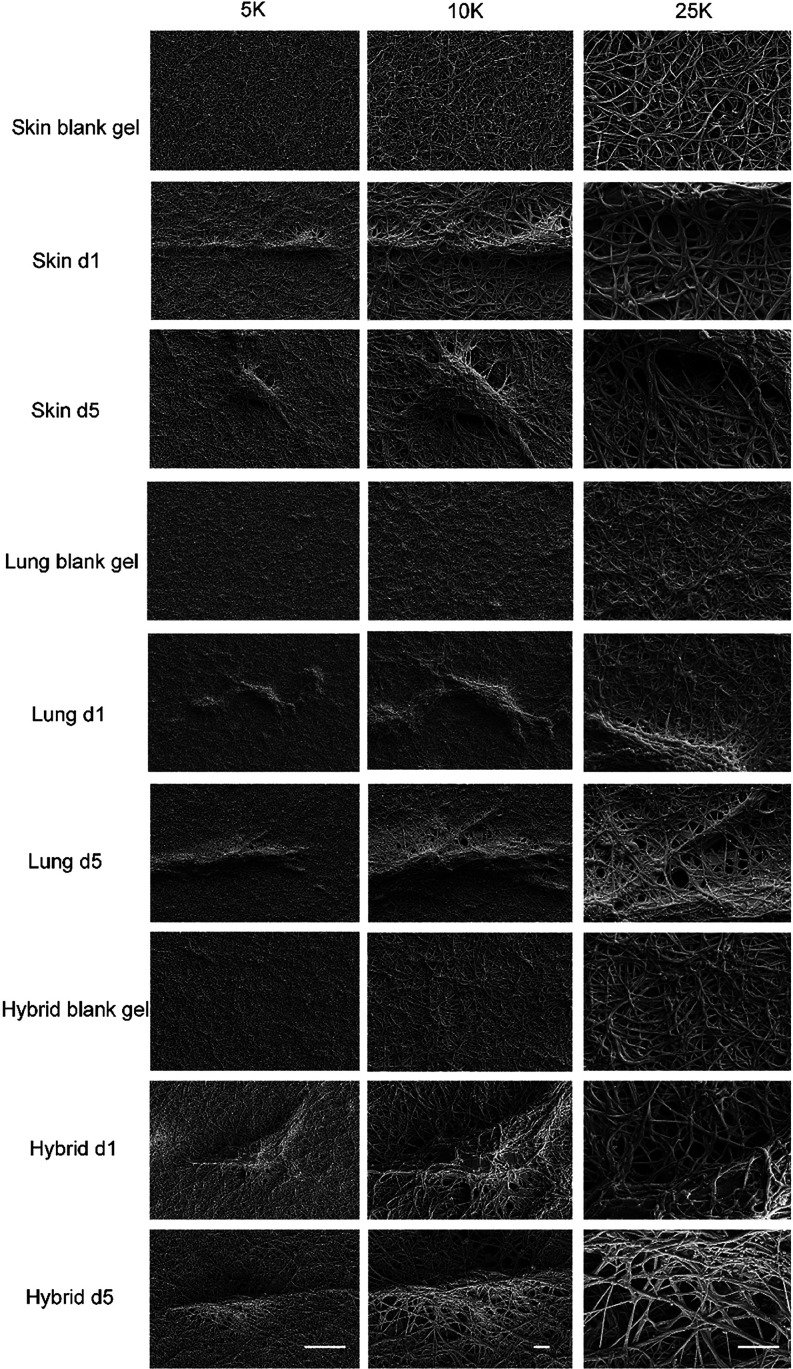
Ultrastructure of the extracellular matrix. Fibers of the matrix in skin, lung, and hybrid ECM hydrogels and fibers of three distinct types of hydrogels loaded with HPEMC at days 1 and 5 at three different magnifications: 5, 10, and 25K. Scale bars represent 10 μm in 5K. Scale bars represent 2 μm in 10 and 25K.

The ultrastructure of the hydrogel was examined by using SEM (Figure 4). Irrespective of the presence of cells, skin, lung, and hybrid ECM hydro each showed a distinct network of erratically organized fibers that were discernible even at lower magnifications (“5K”, Figure 4). The fiber mesh in the lung ECM hydrogel exhibited a notably denser configuration, characterized by numerous fine pores, in contrast to the skin and hybrid ECM hydrogels. Among these, the pores within the fiber network of the skin ECM hydrogel were the largest, while those in the hybrid ECM hydrogel were intermediate in size. Following the seeding of cells within all types of ECM hydrogels, the matrix surrounding HPMEC underwent reorganization, characterized by the noticeable thickening of fibers and the formation of pores.

**Figure 5:**
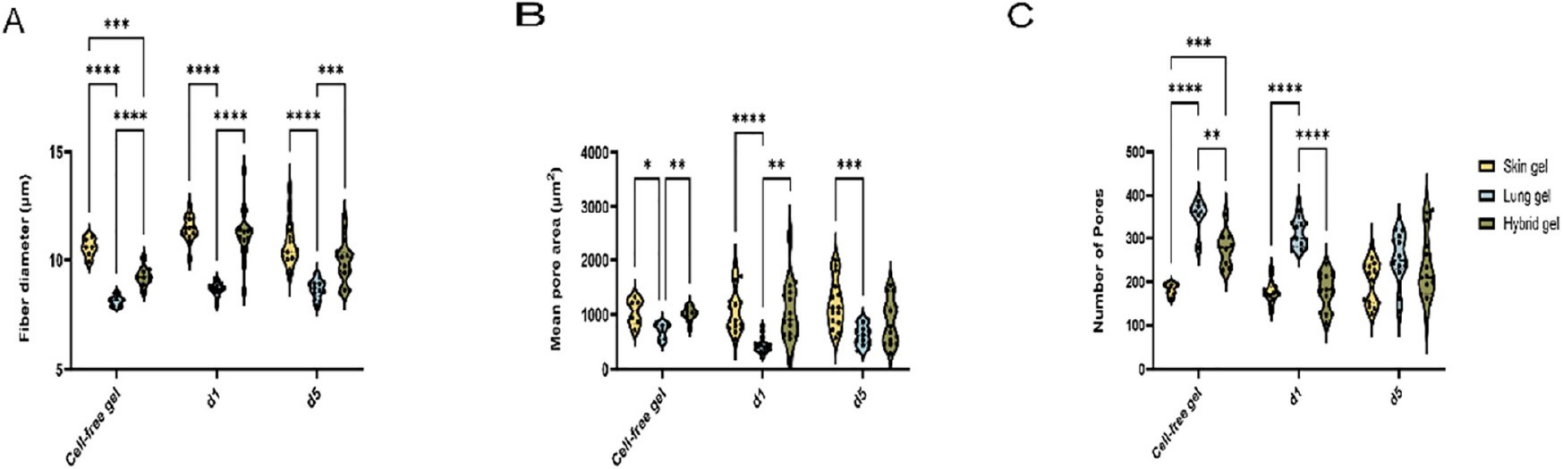
Analyses of the microstructure of the fibers and pores. (A) Mean fiber diameter of cell-free skin, lung, and hybrid ECM hydrogel and Mean fiber diameter of HPMEC-loaded skin, lung, and hybrid ECM hydrogel at days 1 and 5. (B) Mean pore area within the fiber mesh of cell-free skin, lung, and hybrid ECM hydrogels. Mean pore area of HPMEC-loaded skin, lung, and hybrid ECM hydrogels at days 1 and 5. (C) Number of pores within the fiber mesh of cell-free skin, lung, and the hybrid ECM hydrogel. The number of pores within the fiber mesh of HPMEC-loaded skin, lung, and hybrid ECM hydrogels at days 1 and 5.

By analyzing the fibers surrounding the cells, the average fiber diameter in lung ECM hydrogels was calculated; this was always smaller than that in skin or hybrid ECM hydrogels, irrespective of the presence of HPMEC (Figure 5A). Specifically, the average diameter of the fibers in cell-free skin ECM hydrogels was larger than in lung ECM hydrogels (Figure 5A, 10.57 ± 0.42 vs 8.128 ± 0.2299, *p* < 0.0001). Additionally, the average diameter of the fibers in the hybrid ECM hydrogel was intermediate between those of skin and lung ECM hydrogels (Figure 5A). Furthermore, the diameter of fibers in the skin and hybrid ECM hydrogels remained larger than that of the lung ECM hydrogel on both day 1 and day 5 (Figure 5A, skin vs lung ECM hydrogel on day 1:11.5 ± 0.6 vs 8.6 ± 0.3, *p* < 0.001; on day 5:10.8 ± 1.1 vs 8.7 ± 0.4, *p* < 0.001; hybrid vs lung ECM hydrogel on day 1:11.2 ± 1.2 vs 8.6 ± 0.3, *p* < 0.001; on day 5:10.0 ± 1.0 vs 8.7 ± 0.4, *p* < 0.001). Comparing the HPMEC-loaded ECM hydrogel to the cell-free ECM hydrogel, the fibers in the HPMEC-loaded ECM hydrogel showed increased diameter at day 1, regardless of the gel type (Suppl. Figure S3A, *p* < 0.001). At day 5, the fibers’ diameter in the lung ECM hydrogel remained thicker compared to cell-free lung ECM hydrogel, whereas the diameter of the fibers in the skin and hybrid ECM hydrogels did not show differences compared to the respective cell-free ECM hydrogels. The fiber diameters of the ECM hydrogel proximal to the HPMEC did not differ from fibers distal to the HPMEC after 5 days (Suppl. Figure S3D).

The size (Figure 5B) and number of pores (Figure 5C) depended on the presence of cells as well as the ECM origin. The average size of the “mesh holes” between fibers (mean pore area) was larger in cell-free skin ECM hydrogel and hybrid ECM hydrogel compared to the lung ECM hydrogel (Figure 5B, skin ECM hydrogel vs lung ECM hydrogel: 1046 ± 240.4 vs 702.9 ± 142.9, *p* < 0.001; hybrid ECM hydrogel: lung ECM hydrogel: 1006 ± 131.0 vs 702.9 ± 142.9, *p* < 0.001). The pore areas in the skin ECM hydrogels and hybrid ECM hydrogels were larger than those in the lung ECM hydrogels on day 1 (Figure 5B). This disparity with lung ECM hydrogels persisted even after 5 days of HPMEC culturing in skin ECM hydrogels (Figure 5B, 1197 ± 448.9 vs 622.3 ± 190.1, *p* = 0.0007), yet the mean pore area of lung ECM hydrogels had increased by day 5 to similar levels as hybrid ECM hydrogels (Figure 5B). Pore areas of seeded skin and hybrid ECM hydrogels did not differ upon culturing for one and 5 days compared to the cell-free ECM hydrogel (Suppl. Figure S3B). Intriguingly, on day 1, the pore area in HPMEC-seeded lung ECM hydrogels was smaller than cell-free ECM hydrogels and day 5 gels (Suppl. Figure S3B). Also, the pore area of the hybrid ECM hydrogel proximal to the HPMEC was larger than it was distal to the HPMEC after 5 days (Suppl. Figure S3E).

In contrast, the number of pores in the skin ECM hydrogel was less than in the lung ECM hydrogel (Figure 5C, 182.0 ± 13.7 vs 353.2 ± 38.6, *p* < 0.0001) and also lower than in the hybrid ECM hydrogel (182.0 ± 13.7 vs 277.7 ± 40.94, *p* = 0.0002). While the differences in the number of pores were maintained at 1 day postseeding, the average number of pores in the skin and hybrid ECM hydrogels had increased at day 5 postseeding and were similar to lung ECM hydrogels (Figure 5C). Furthermore, the number of pores in HPMEC-seeded skin ECM hydrogels did not change compared to cell-free ECM hydrogels at days one and five (Suppl. Figure S3C). In contrast, the number of pores had decreased on day 1 in HPMEC-seeded lung ECM hydrogels compared to the cell-free lung ECM hydrogels (*p* = 0.0465), and this decrease continued at day 5 (*p* = 0.0107) compared to day 1. Similarly, the number of pores had decreased in HPMEC-seeded hybrid ECM hydrogels at day 1 (*p* = 0.0016). The number of pores in the skin ECM hydrogel and hybrid ECM hydrogel remained lower than that in the lung ECM hydrogel after HPMEC seeding on day 1 (Figure 5C, skin vs lung ECM hydrogel: 175.9 ± 25.8 vs 314.3 ± 35.4, *p* < 0.001; hybrid vs lung ECM hydrogel: 179.1 ± 42.2 vs 314.3 ± 35.4, *p* < 0.001). The number of pores around the HPMEC was higher than those distal from the HPMEC in the lung ECM hydrogels after 5 days of culturing (Suppl. Figure S3F, *p* = 0.00211). However, regarding the proliferation of HPMEC and remodeling of ECM, no differences were observed among the three gels on day 5 (Figure 5C).

The ultrastructure analyses revealed that the hybrid ECM hydrogels exhibited a composite architecture, representing a combination of skin and lung ECM hydrogels. ECs change the architecture of their 3D microenvironment over time. The differences in fiber diameter and pore area persisted among the three gels notwithstanding a reduction of these differences by endothelial cell activities.

The data are from three independent experiments. Five randomly selected ROIs were measured for every single sample, each dot represents a measurement of a randomized region. Tukey’s multiple comparisons test, *p < 0.05, **p < 0.01, ***p < 0.001.

### Degradation of Hydrogels Is Organ-Dependent

**Figure 6:**
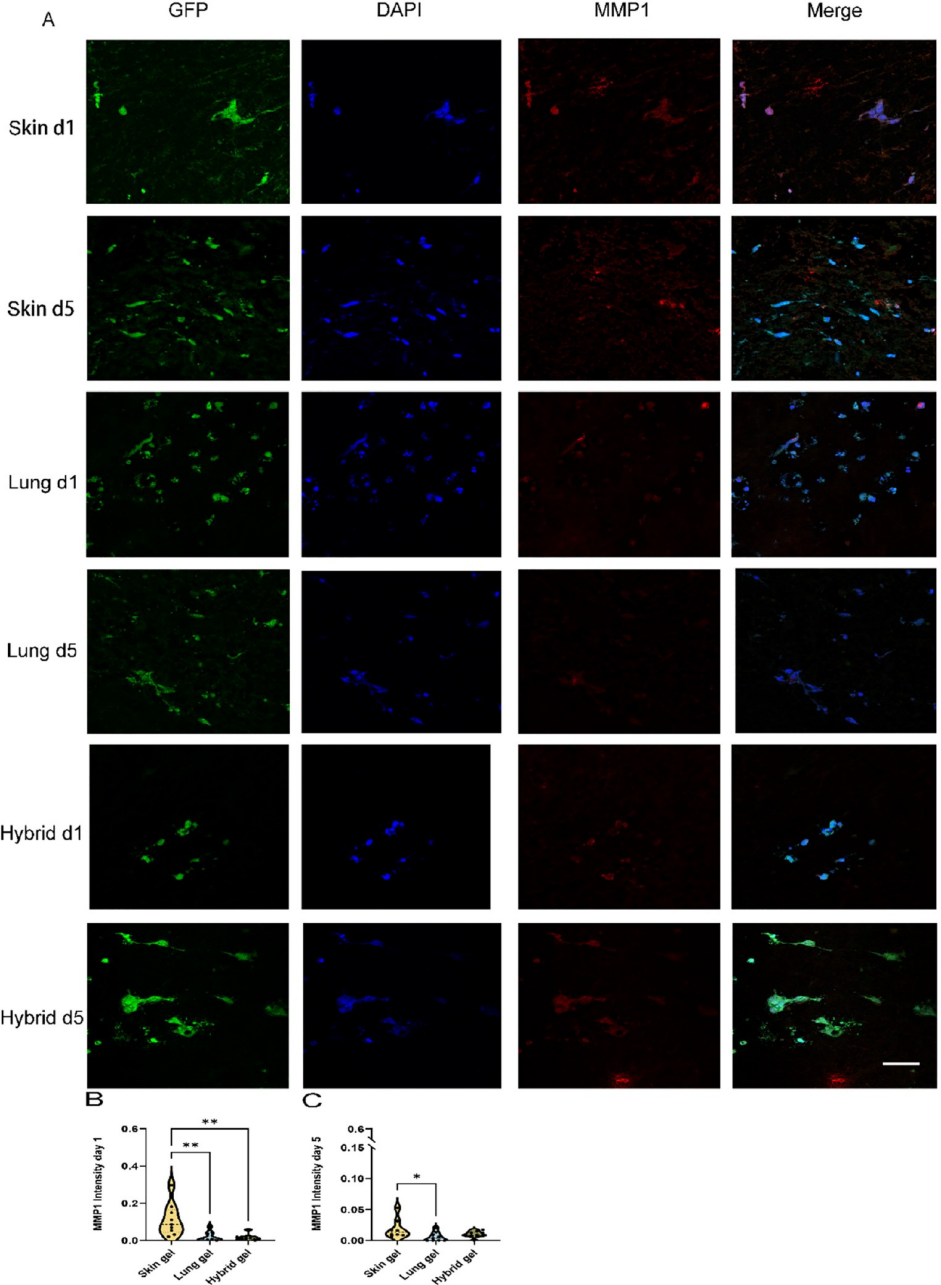
Fluoromicrographs of MMP1 staining. (A) Representative images of fibronectin staining of 4 μm sections of paraffin-embedded hydrogels. Merged images: green, GPF-labeled HPMEC; red, MMP1; blue, nuclei (DAPI). Scale bar: 58 μm. (B) Comparison of the MMP1 intensity per nuclei among three different gels at day 1. (C) Comparison of MMP1 intensity per nuclei among three different gels at day 5.

To investigate whether the changes in pore area and number were attributed to the degradation of the ECM hydrogel by HPMEC during VNF, we examined the presence of archetype ECM-degrading proteases MMP1 and MMP9 by immunofluorescent staining. Cell-free ECM hydrogels were devoid of detectable levels of MMP1 (Suppl. Figure 4). During HPMEC VNF on day 1 and day 5, no more than minimal MMP1 expression was observed in the lung and hybrid ECM hydrogels (Figure 6A, red). In contrast, the skin ECM hydrogel showed significant MMP1 deposition as early as after 1 day of culturing (Figure 6B), and its expression remained higher than that in the lung ECM hydrogel after 5 days (Figure 6C, 0.0189 ± 0.0158 vs 0.00583 ± 0.00719, *p* = 0.0363). Interestingly, the differences in MMP1 expression between the skin ECM hydrogel and hybrid ECM hydrogel on day 1 (Figure 6B) disappeared after 5 days (Figure 6C). MMP1 was predominantly localized around cells, with scattered deposition more distally in the hydrogel.

The data are from three independent experiments. Three different randomized regions were measured for every single sample, and each dot represents a measurement of a randomized region. One-way ANOVA comparing gel, *p < 0.05, **p < 0.01.

**Figure 7:**
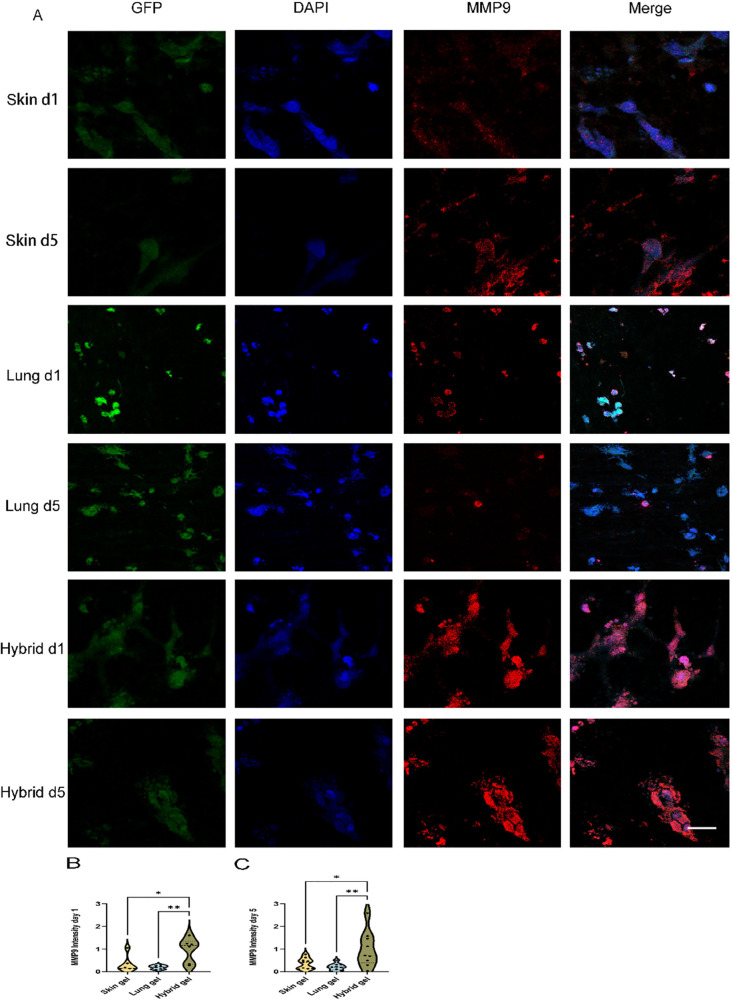
Fluoromicrographs of MMP9 staining. (A) Representative images of fibronectin staining of 4 μm sections of paraffin-embedded hydrogels. Merged images: green, GPF labeled HPMEC; red, MMP9; blue, nuclei (DAPI). Scale bar: 58 μm. (B) Comparison of the MMP9 intensity per nuclei among three different gels at day 1. (C) Comparison of MMP9 intensity per nuclei among three different gels at day 5.

Additionally, MMP9 was assessed by immunofluorescent staining. In contrast to MMP1, cell-free lung (and hybrid) ECM hydrogels revealed minimal staining of MMP9 (Suppl. Figure 5). To ensure standardization, the expression level of MMP9 in the HPMEC-loaded hydrogel was normalized against the expression level of MMP9 in the cell-free gel, as described in the methods section. The localization of MMP9 was observed not only in the proximity of HPMEC but also in distal areas of the ECM hydrogel (Figure 7A, red). Intriguingly, the deposition of MMP9 in the hybrid ECM hydrogel did not exhibit an intermediate level between the skin and lung; instead, its expression was higher than both the deposition in the skin ECM hydrogel and the lung ECM hydrogel on day 1 (Figure 7B) and day 5 (Figure 7C).

The data are from three independent experiments. Three randomly selected ROIs were measured for every single sample, and each dot represents a measurement of a randomized region. One-way ANOVA comparing gel, *p < 0.05, **p < 0.01.

### VNF Directs Novel Deposition of Vasculogenic ECM Components in an Organ-Dependent Fashion

**Figure 8:**
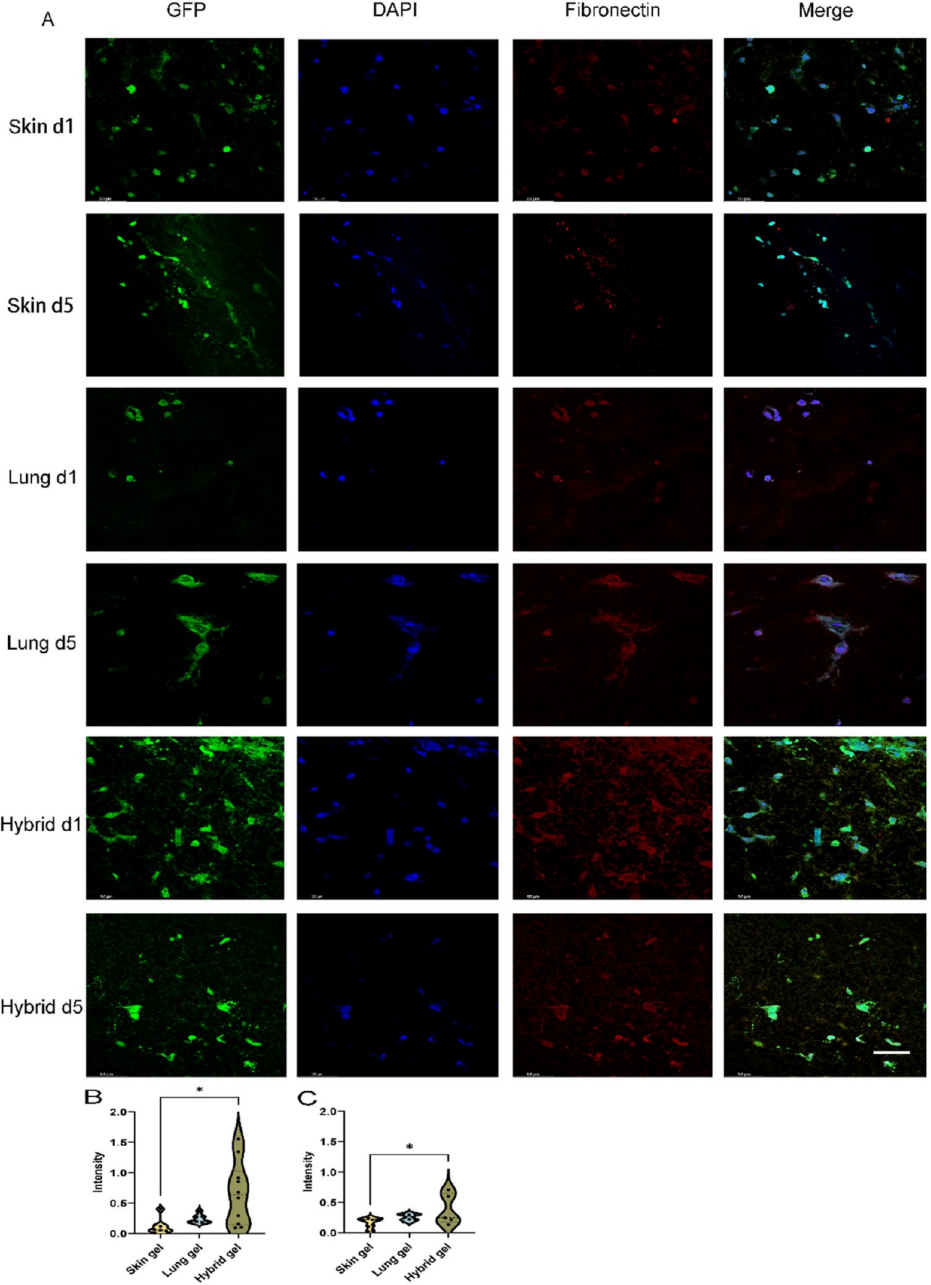
Fluoromicrographs of fibronectin-staining. (A) Representative images of fibronectin staining of 4 μm sections of paraffin-embedded hydrogels. Merged images: green, GPF-labeled HPMEC; red, fibronectin; blue, nuclei (DAPI). Scale bar: 58 μm. (B) Comparison of the fibronectin intensity per nuclei among three different gels at day 1. (C) Comparison of fibronectin intensity per nuclei among three different gels at day 5.

All three types of cell-free ECM hydrogels contained no detectable fibronectin (Suppl. Figure 6). After 1 day of culturing, HPMEC-seeded hybrid ECM hydrogels showed a strong deposition of fibronectin (Figure 8A), which remained on day 5. In contrast, only minimal fibronectin deposition had occurred in the skin and lung ECM hydrogels at days one and five. The cross-sectional fluorescence intensity on days 1 and 5 was analyzed by CellProfiler (Figure 8B,C). The expression of fibronectin in hybrid ECM hydrogel was higher than in skin ECM hydrogel on day 1 (Figure 8B, 0.658 ± 0.516 vs 0.118 ± 0.132, *p* = 0.0128) and day 5 (Figure 8C, 0.381 ± 0.255 vs 0.164 ± 0.0831, *p* = 0.0247).

The data are from three independent experiments. Three randomly selected ROIs were measured for every single sample, and each dot represents a measurement of a randomized region. One-way ANOVA comparing gel, *p < 0.05.

**Figure 9:**
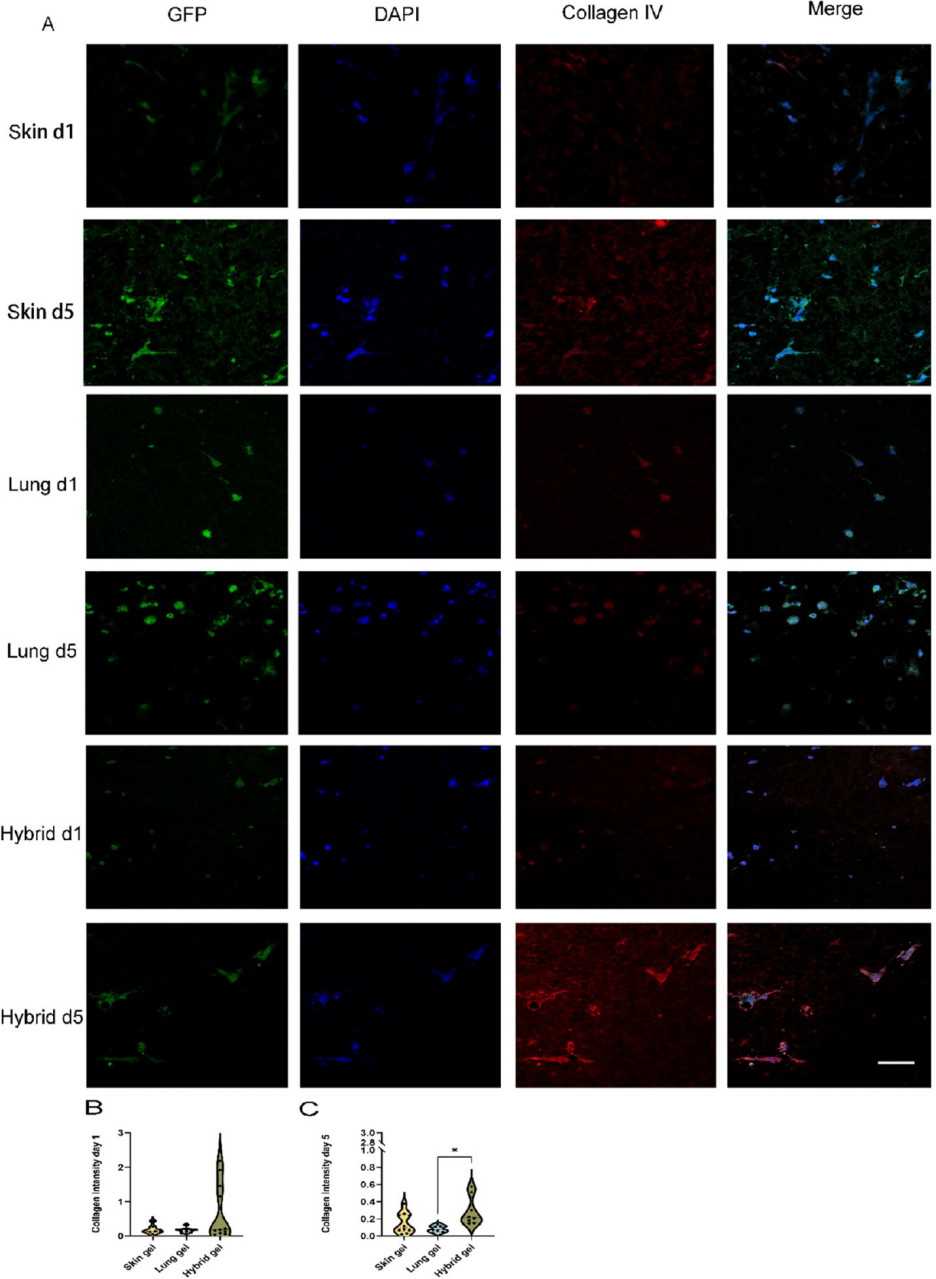
Fluoromicrographs of collagen IV staining. (A) Representative images of fibronectin staining of 4 μm sections of paraffin-embedded hydrogels. Merged images: green, GPF-labeled HPMEC; red, collagen IV; blue, nuclei (DAPI). Scale bar: 58 μm. (B) Comparison of collagen IV intensity per nuclei among three different gels at day 1. (C) Comparison of collagen IV intensity per nuclei among three different gels at day 5. The data are from 3 independent experiments. Three randomly selected ROIs were measured for every single sample, and each dot represents a measurement of a randomized region. One-way ANOVA comparing gel, *p < 0.05.

Besides fibronectin, the basement membrane constituent collagen IV is also involved in the vascularization processes. The immunostained fluoromicrographs showed that collagen IV had a deposition pattern similar to that of fibronectin. Cell-free hydrogels harbored negligible amounts of collagen IV (Suppl. Figure 7). The deposition of collagen IV by HPMEC was reminiscent of fibronectin (Figure 9A–C). Again, at days one and five, minimal deposition had occurred in skin and lung ECM hydrogels while hybrid gels showed a high deposition compared to lung ECM hydrogels on day 5 (Figure 9C, 0.286 ± 0.167 vs 0.082 ± 0.039, *p* = 0.0110).

An overview of the measurements made throughout this section is provided in Table 3.

**Table 3:** Summary of the Measurements of All Parameters in the Three Hydrogels on Day 1 and Day 5^a^

features	skin ECM hydrogel		lung ECM hydrogel		hybrid ECM hydrogel	
time point	day 1	day 5	day 1	day 5	day 1	day 5
stiffness	+	+	+++	+	++	+
total stress relaxation	+	+	+	+	+	++
fiber diameter	++++	++++	+	++	+++	+++
mean pore area	++++	++++	+	++	+++	+++
number of pores	++	++	+++	++	++	++
Ki67	+++	++	++	+	++	++
MMP 1	+++	++	+	+	+	++
MMP9	++	++	+	+	+++	+++
fibronectin	+	+	+	+	++	+
collagen IV	+	+	+	+	+	++

a The more + marked, the higher the value recorded.

### Principal Component Analyses

**Figure 10:**
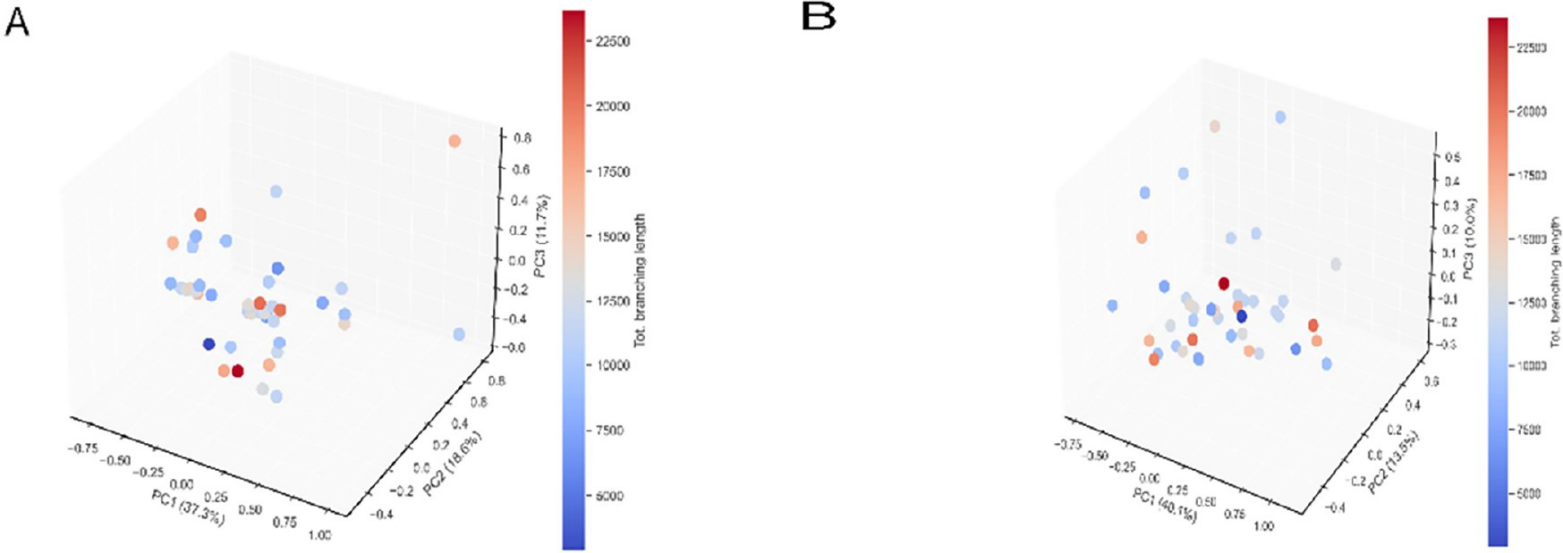
PCA of the features in three distinctive gels. (A) PCA projection onto 3D space of the data set from PCA analysis on day 1. (B) PCA projection onto 3D space of the data set from l PCA analysis on day 5. The colors of the data points represent the number of VNF.

To further understand the interrelationship between, on the one hand, VNF and, on the other hand, culture times, cellular responses, and matrix changes, we performed a principal component analysis (PCA). In these analyses, we did not take into consideration the different types of hydrogels. Principal components that defined the VNF-dependent variable “total branching length” were analyzed and 3D plotted. The components of gels and cell responses were categorized at two time points: day 1 and day 5. On day 1, there was no evident clustering of data points observed between the variables measured in this study (Figure 10A). In contrast, the 3D PCA scatter plots on day 5 showed distinct and discernible clustering (Figure 10B), which indicated a strong correlation between the PCA-derived component groups and the VNF. We further dissected the PCA clusters to reveal hitherto unknown relationships between the parameters we had measured and VNF. We elected to examine the number of principal components that collectively accounted for a minimum of 60% of the cumulative variance within the data. Upon inclusion of the third principal component, as shown in the scree plots (Suppl. Figure 8), the cumulative proportion of variance explained exceeded the threshold of 60%. The relative influence of the three principal components (PC1, PC2, and PC3) on day 5 individually accounted for 40.1, 13.5, and 10.0% of the data description.

Table 4 presents the correlations or weights of the parameters we measured with each principal component. We selected the average arbitrary value of PC at ±0.21365 as the threshold for assessing the contribution of features in relation to VNF. Given the samples with longer total branching lengths were generally clustering at the back left-hand quadrant of the 3D plot (negative arbitrary values on PC1 and PC3 but positive arbitrary values on PC2 axes) (dots with red shades), we standardized all components as described in methods for easier understanding. Features with contributions of more than 0.21365 arbitrary units were interpreted as having a strong positive impact on VNF, whereas those less than −0.21365 arbitrary units were construed as needing to be reduced to allow VNF. In PC1 PCA indicated a lower stiffness, slim fiber diameter with small but plentiful pores was the ideal environment for VNF. In PC2 the combination of lower stiffness, increased stress relaxation, a reduced number of pores, and enhanced presence of collagen IV supported VNF. In PC3 the less presence of collagen IV, MMP1, and MMP9 with slim fiber diameters and reduced total stress relaxation with endothelial cells that had a lower rate of proliferation supported enhanced VNF.

**Table 4:** Contributions of the Measured Variables within Each Principal Component Group^a^

feature	PC1	PC2	PC3
stiffness	–0.3762	–0.3143	
total stress relaxation		0.7496	0.2517
fiber diameter	–0.4656		–0.2198
mean pore area	–0.5192		
number of pores	0.4788	–0.2913	
Ki67 ratio	–0.2686		–0.3100
MMP 1			–0.2731
MMP9			–0.4526
collagen IV		0.3685	–0.6657

a Units are arbitrary units.

## Discussion

In this study, we examined the impact of various organ-derived hydrogels on the VNF by ECs. We found that skin ECM-derived hydrogels are the most potent enhancers of VNF and endothelial cell proliferation compared to ECM-derived hydrogels from the lung or a hybrid of both ECM sources. Our second main finding was that mechanical properties of the hydrogels influenced VNF and subsequent ECM remodeling in a time- and organ (mixture)-dependent fashion. Principal mechanical properties of the gel including stiffness, viscosity, the number and the area of the pores, and diameter exert a more pronounced influence on the VNF than the biochemical proteins such as MMP9, fibronectin, and collagen IV. Finally, hybrid ECM hydrogels did not influence VNF or associated processes in a manner that mirrors the “average” effects observed with the skin and lung ECM hydrogels.

The process of vascularization involves a complex interaction between ECs and the ECM environment. Underlying mechanisms remain elusive. The morphology of biomaterial fibers influences cell adhesion, proliferation, and orientation.^26^ Larger pores affect cell seeding, distribution, migration, and further neovascularization in vivo.^27−29^ Our research facilitated the evaluation of the mutual interactions between ECs and the ECM across a span of time. PCA indicated that more small pores within the fiber matrix contributed to the enhancement of the VNF when the fibers were thinner. An optimal ECM architecture is one characterized by pores that are sufficiently spacious to facilitate easy cell penetration into the internal spaces, while simultaneously preventing excessive pore size that might hinder cells from effectively extending and stretching between the fibers.^30^ Our PCA results were in alignment with this finding. An adequate number of pores within the ECM hydrogel, serving as a positive factor, offered ample space for cell proliferation, thereby promoting VNF. Conversely, when the pore areas were excessively large, the ECs were unable to establish connections with one another, thus acting as a negative factor inhibiting VNF in our study.

The ultrastructure of the ECM hydrogel also impacted the proliferation of ECs.^31^ PCA revealed that a reduced Ki67, indicative of proliferation, stood out as another primary determinant of VNF on day 5. The lower proliferation of HPMEC in the lung and hybrid ECM hydrogels as compared to skin ECM hydrogels was beneficial for VNF on day 5, whereas excessive proliferation within the skin ECM hydrogel led to the cellular occupation of pores among the ECM hydrogel fibers, potentially limiting VNF.

Understanding the influence of ECM stiffness on ECs holds significant importance.^32^ In collagen or fibrin hydrogels or on ECM-coated polyacrylamide gels, an increased stiffness of the ECM hampered VNF,^33^ resulting in shorter, thicker, slower-growing sprouts, fewer branching points, and reduced network connectivity.^34−36^ Nonetheless, this stiffness-induced effect also promotes the formation of larger and more stable lumens.^37,38^ Our results of native organ-derived ECM hydrogels corroborate these findings. The stiffer lung ECM hydrogels had less VNF than the skin ECM hydrogels. Moreover, as per the results of the PCA, it was found that the stiffness of gels on day 5 was a strong regulatory factor, with reduced stiffness promoting VNF in both PC1 and PC2.

The architectural and stiffness changes during VNF are mediated through matrix remodeling, as well as through pulling forces that are exerted by the cells on the fiber networks. During wound healing and the associated vascularization processes, ECM remodeling is a dynamic, spatiotemporally regulated process. Remodeling of ECM during vascularization is a balance of degradation by MMPs^39,40^ and deposition of vasculogenic ECM components, like fibronectin and collagen IV. Our results show that MMPs are upregulated in the ECM during VNF and may contribute to decreases in stiffness that negatively correlate with VNF. The stiffness of hybrid ECM hydrogel decreased after 5 days of culturing, corresponding with the highest expression of MMP9 among the three types of gels. The gelatinase MMP9 can degrade collagen IV^41^ which was highly expressed in the hybrid ECM hydrogel. These findings suggested that a regulatory feedback loop exists between the expression levels of MMP9 and collagen IV in hybrid ECM hydrogels. Importantly, this shows that ECs in hybrid ECM hydrogels do not respond merely as the average responses of ECs in both individual gels but rather have a defined response to this unique environment.

MMP1-mediated ECM degradation facilitated the VNF of ECs.^42^ MMP1 expression in skin ECM hydrogels was highly expressed compared with the other gels. According to the outcomes derived from PCA, MMP1 was identified as one of the contributing features that augments VNF in PC3. We assume that highly expressed MMP1 was the consequence of over-proliferation of the ECs. During VNF when ECs needed more space to build the branches and mesh within the hydrogels, they facilitated this through the expression of MMPs. Regarding fibronectin, there was a more dispersed distribution and a higher level of expression in the hybrid ECM hydrogels. Fibronectin appeared to have supported cell adhesion of ECs during VNF in skin and lung ECM hydrogels, while in hybrid ECM hydrogels, it may also have mainly acted to stabilize the ECM by bridging collagen fibers.^43^ The extent of the contribution of the deposition of fibronectin to the VNF formation requires more investigation, as it did not feature in our PCA analyses. In terms of biochemistry, the hybrid ECM hydrogel did not demonstrate an intermediary behavior between the skin and lung ECM hydrogels, despite its physical properties (stiffness, viscosity, fiber diameter, pore size, and number of pores) falling between those of the skin and lung ECM hydrogels.

In this research, PCA was performed on the ECs phenotype and ECM property data set to draw correlations between ECs response and ECM properties to establish correlations between ECs responses and ECM attributes. PCA stands as a powerful method to predict the significance of individual features within a multifactorial biomaterial affecting VNF. After analyzing 10 features including physical characteristics (viscoelasticity and architecture) and biochemical features pertaining to ECM turnover, the physical properties were shown to influence the VNF more than the biochemical properties. However, we have not yet investigated the specific influence of distinct chemical components, such as collagen I and GAGs, originally present in the three different gels, nor did we consider the individual compositions of the three different hydrogels in these analyses. Also, it is challenging to definitively attribute the lower significance of biochemical proteins secreted by ECs, such as MMPs and fibronectin, to VNF, as it remains unclear whether this outcome is a consequence of the physical properties or if physical properties indeed play a more dominant role in VNF. The therapeutic ramifications of our findings are that by mixing different organ-derived ECM hydrogels it is possible to “tweak” its influence on vascularization.

## Conclusions

This study shows that VNF by HPMEC in ECM hydrogels or mixtures depends on the organ origin of the ECM. The mutual interaction showed that the initial physical properties of the gels influence VNF while at later stages, cellular-driven changes in gels’ physical properties also influence the process.

## Supporting Information Available

Dissection of endothelial branching networks, physical properties of the ECM hydrogels and porosities, immunofluorescent staining for MMPs, and ECM components (PDF)
