# Supplementary material for: Physical Properties and Biochemical Composition of Extracellular Matrix-Derived Hydrogels Dictate Vascularization Potential in an Organ-Dependent Fashion
Source: ACS Appl Mater Interfaces. 2024 May 31;16(23):29930–45. doi: 10.1021/acsami.4c05864 (PMC11181272; doi:10.1021/acsami.4c05864)
Supplement: Supplementary file 1 — am4c05864_si_001.pdf [file am4c05864_si_001.pdf]

Supporting Information

Physical properties and biochemical composition of extracellular matrix-derived hydrogels dictate vascularization potential in an organ-dependent fashion.

Meng Zhang [1,2], Fenghua Zhao [2,3], Yuxuan Zhu[4], Linda A. Brouwer [1], Hasse Van der Veen[1], Janette K. Burgess [1,2,5*] and Martin C. Harmsen [1,2, 5*]

1 University of Groningen, University Medical Center Groningen, Department of Pathology and Medical Biology, Hanzeplein 1 (EA11), 9713 GZ Groningen, The Netherlands

2 University of Groningen, University Medical Center Groningen, W.J. Kolff Institute for Biomedical Engineering and Materials Science-FB41, A. Deusinglaan 1, 9713 AV Groningen, The Netherlands

3 University of Groningen, University Medical Center Groningen, Department of Biomedical Engineering-FB40, A. Deusinglaan 1, 9713 AV Groningen, The Netherlands

4 Department of Computer Science, Rensselaer Polytechnic Institute, Troy, NY 12180, USA

5 University of Groningen, University Medical Center Groningen, Groningen Research Institute for Asthma and COPD (GRIAC), Hanzeplein 1 (EA11), 9713 AV Groningen, The Netherlands

*Correspondence: Prof. Dr. Martin C. Harmsen (* m.c.harmsen@umcg.nl)
Prof. Dr. Janette K. Burgess (* j.k.burgess@umcg.nl)

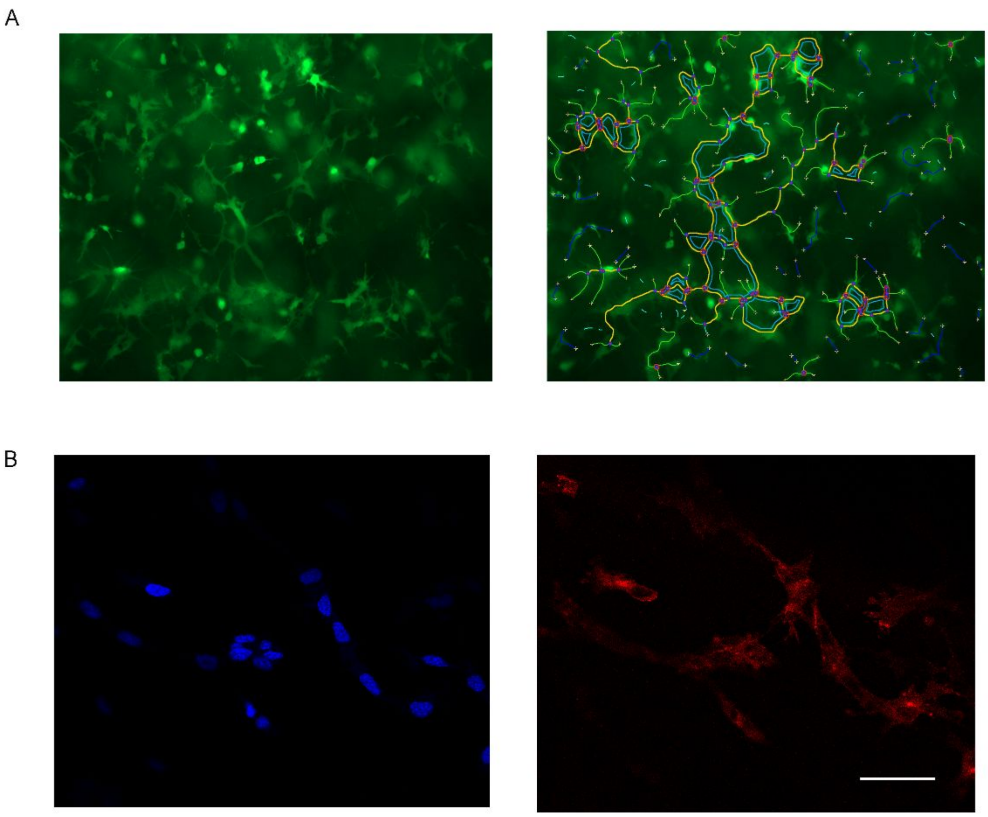

Figure S1

Fig.S1. Initial image enlargement of the network formed by HPMEC was analyzed by endothelial tube formation assays. (A) Branches are denoted in green, segments in magenta, junctions are encapsulated in red and surrounded by blue, meshes are indicated in cyan, and the master tree, composed of segments linked by master junctions, is depicted in yellow. (B) VE-cadherin staining in for the endothelial markers. Red – VE-cadherin, blue – nuclei (DAPI). Scale bar: 58 μm.

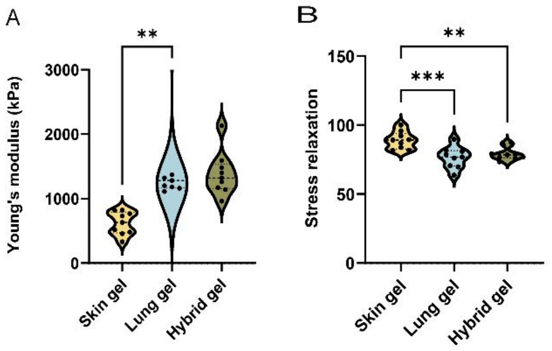

Figure S2

Fig.S2. Comparison of physical characteristics of three distinct types of cell-free ECM hydrogels. (A) Stiffness of skin, lung, and hybrid ECM hydrogel. (B) Total stress relaxation of skin, lung, and hybrid ECM hydrogel.

The data are from three independent experiments. Three randomly selected ROIs were measured for every single sample, and each dot represents a measurement of a randomized region. one-way ANOVA comparing gel, ** p< 0.01, **** p <<0.0001.

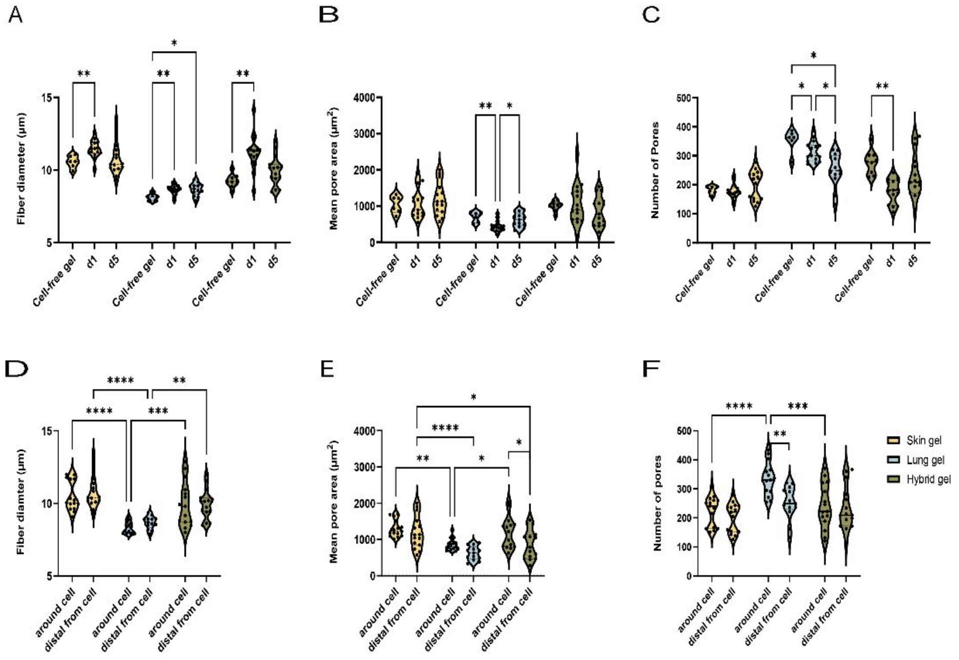

Figure S3

Fig.S3. Comparison of the microstructure of the fibers and pores. (A) Comparison of mean fiber diameter among cell-free ECM hydrogel, and HPMEC-loaded skin at day 1 and 5 in skin, lung and hybrid ECM hydrogel respectively. (B) Comparison of mean pore area among cell-free ECM hydrogels, and HPMEC-loaded hydrogels at day 1 and 5 culture in skin, lung, and hybrid ECM hydrogels respectively. (C) Comparison of the number of pores within the fiber mesh among cell-free ECM hydrogel, and HPMEC-loaded hydrogels at day 1 and 5 in skin, lung, and hybrid ECM hydrogels respectively. (D) Comparison of mean fiber diameter of three distinctive hydrogels around the HPMEC and distal from the HPMEC at day 1 5 days. (E) Comparison of mean pore area of three distinctive hydrogels proximal and distal to the HPMEC at day 1 5 days. (F) Comparison of the number of pores within the fiber mesh of three distinctive hydrogels proximal and distal to the HPMEC at day 1 5.

The data are from 3 independent experiments. 5 randomly selected ROIs were measured for every single sample, each dot represents a measurement of a

randomized region. Tukey's multiple comparisons test, * $p < 0.05$, ** $p < 0.01$,*** $p < 0.001$, **** p <<0.0001.

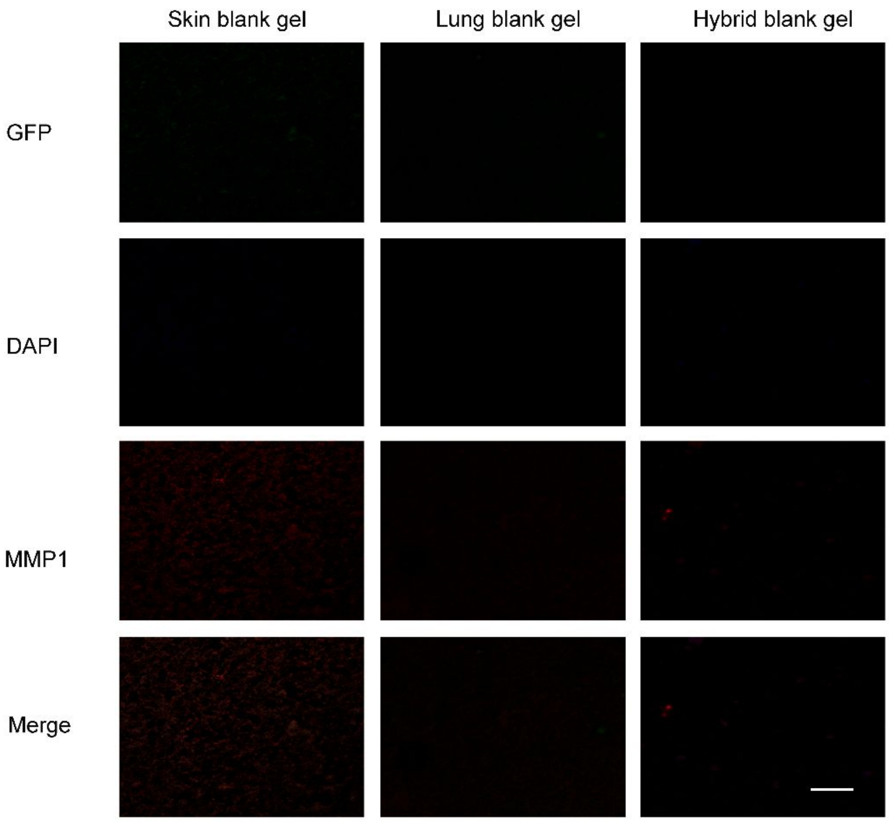

Figure S4

Figure S4. Fluoromicrographs of MMP1 staining of three distinctive cell-free hydrogels. GPF-labeled HPMEC, red – MMP1, blue – nuclei (DAPI). Scale bar: 58 μm.

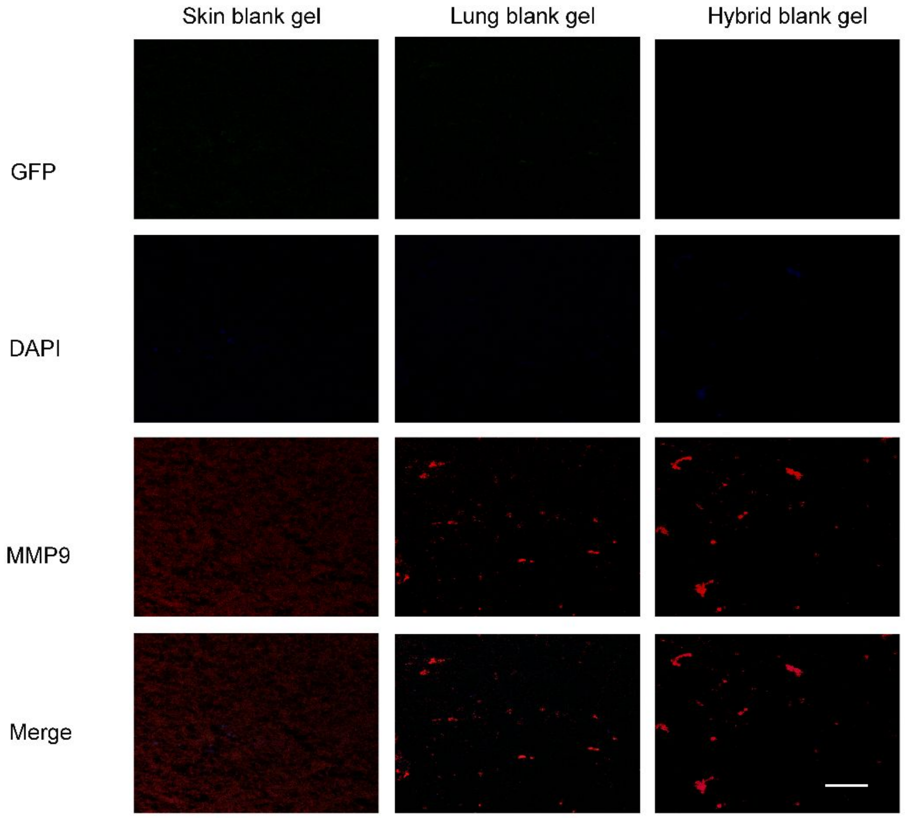

Figure S5

Figure S5. Fluoromicrographs of MMP9 staining of three distinctive cell-free hydrogels. GPF-labeled HPMEC, red – MMP9, blue – nuclei (DAPI). Scale bar: 58 μm.

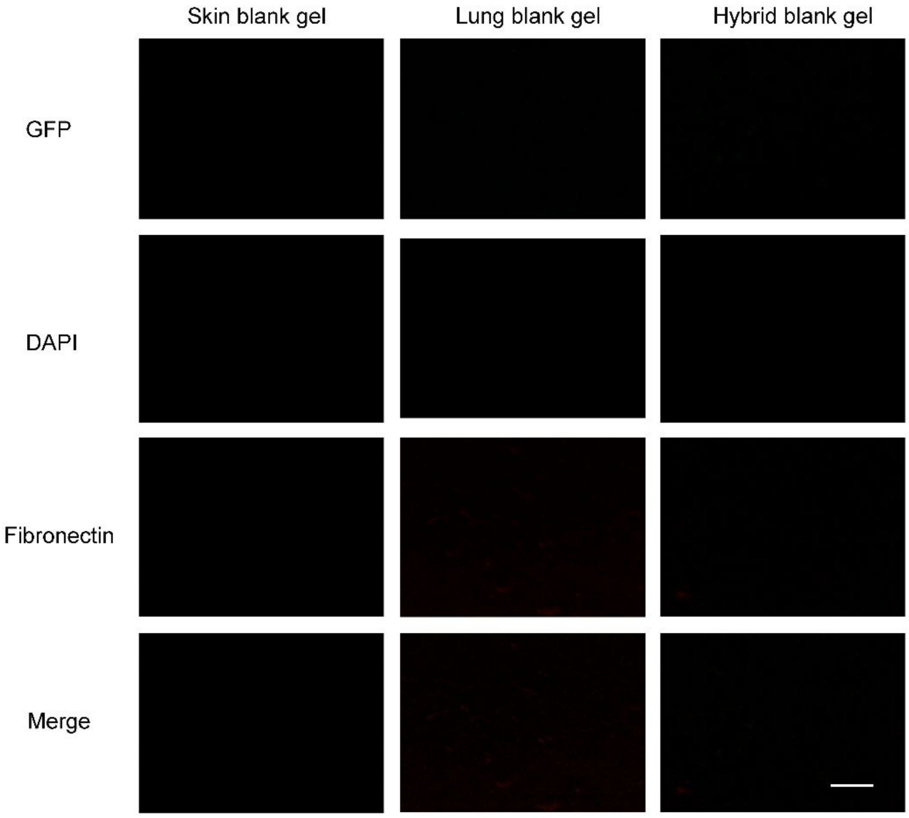

Figure S6

Figure S6. Fluoromicrographs of fibronectin staining of three distinctive cell-free hydrogels. GPF-labeled HPMEC, red – fibronectin, blue – nuclei (DAPI). Scale bar: 58 μm.

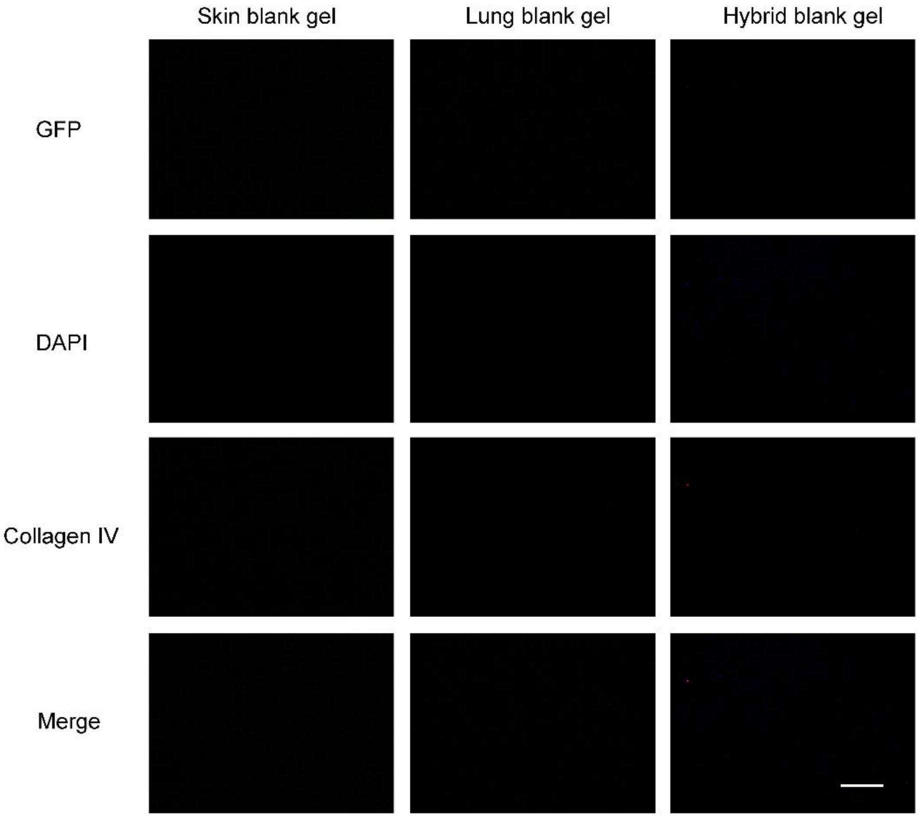

Figure S7

Figure S7. Fluoromicrographs of collagen IV staining of three distinctive cell-free hydrogels. GPF-labeled HPMEC, red – collagen IV, blue – nuclei (DAPI). Scale bar: 58 μm.

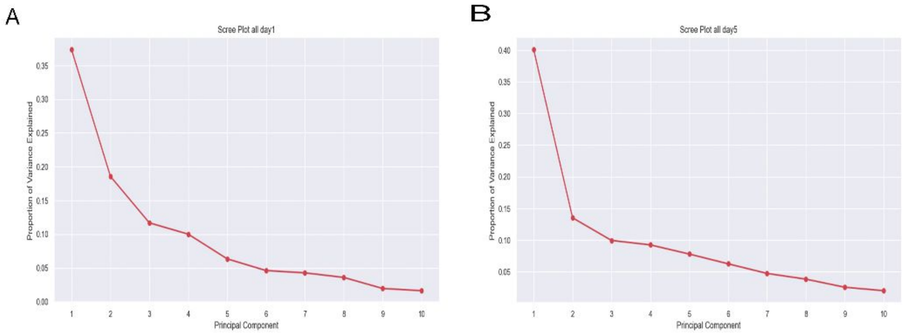

Figure S8

Figure S8. Scree plot representing proportion of principal component within measured features in three type of ECM hydrogels. (A) Scree plot

representing proportion of principal component of three types of hydrogels on day 1. (B) Scree plot representing proportion of principal component of three types of hydrogels on day 5.
